# Dichlorido[3-meth­oxy­methyl-4-phenyl-5-(2-pyrid­yl)-4*H*-1,2,4-triazole-κ^2^ *N* ^1^,*N* ^5^]copper(II)

**DOI:** 10.1107/S160053681103296X

**Published:** 2011-08-27

**Authors:** Shouping Cao, Zuoxiang Wang, Xiaofei Jin

**Affiliations:** a School of Chemistry and Engineering, Southeast University, Nanjing 211189, People’s Republic of China

## Abstract

In the title complex, [CuCl_2_(C_15_H_14_N_4_O)], the Cu^II^ atom possesses a highly distorted square-planar geometry with N—Cu—N and Cl—Cu—Cl angles of 79.86 (8) and 98.65 (3)°, respectively, while the Cl—Cu—N angles fall into two distinct groups with values of 95.26 (6), 98.75 (6), 150.56 (6) and 152.04 (6)°. The pyridyl ring is twisted by 9.4 (2)° with respect to the triazole ring, which is oriented at approximately right angles [84.66 (8)°] with respect to the phenyl ring.

## Related literature

For general background on the coordination chemistry of 1,2,4-triazoles, see: Klingele & Brooker (2003 ▶); Rubio *et al.* (2011 ▶). For the biological activity of triazoles, see: Isloor *et al.* (2009 ▶). For a related structure, see: Ren *et al.* (2006 ▶).
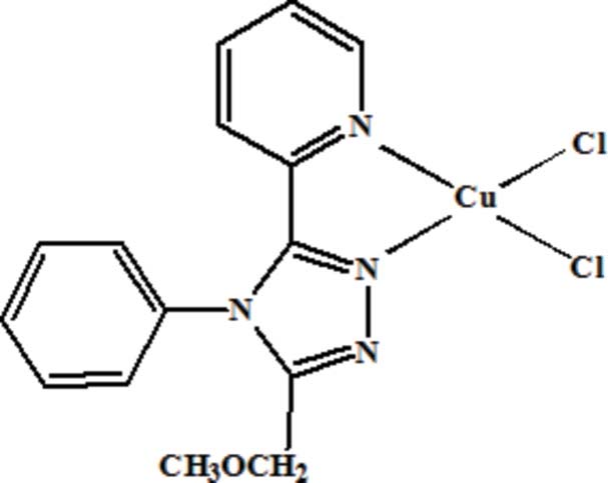


## Experimental

### 

#### Crystal data

[CuCl_2_(C_15_H_14_N_4_O)]
*M* *_r_* = 400.74
Orthorhombic, 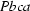

*a* = 16.6512 (11) Å
*b* = 11.2056 (7) Å
*c* = 17.9966 (11) Å
*V* = 3357.9 (4) Å^3^
*Z* = 8
Mo *K*α radiation
μ = 1.63 mm^−1^
*T* = 296 K
0.15 × 0.13 × 0.12 mm

#### Data collection

Bruker APEXII CCD diffractometer
Absorption correction: multi-scan (*SADABS*; Sheldrick, 2003 ▶) *T*_min_ = 0.792, *T*_max_ = 0.829
22829 measured reflections
3043 independent reflections
2288 reflections with *I* > 2σ(*I*)
*R*_int_ = 0.052

#### Refinement

*R*[*F* ^2^ > 2σ(*F* ^2^)] = 0.029
*wR*(*F* ^2^) = 0.068
*S* = 1.00
3043 reflections
210 parameters
H-atom parameters constrained
Δρ_max_ = 0.27 e Å^−3^
Δρ_min_ = −0.30 e Å^−3^

### 

Data collection: *APEX2* (Bruker, 2005 ▶); cell refinement: *SAINT* (Bruker, 2005 ▶); data reduction: *SAINT*; program(s) used to solve structure: *SHELXS97* (Sheldrick, 2008 ▶); program(s) used to refine structure: *SHELXL97* (Sheldrick, 2008 ▶); molecular graphics: *SHELXTL* (Sheldrick, 2008 ▶); software used to prepare material for publication: *SHELXTL*.

## Supplementary Material

Crystal structure: contains datablock(s) I, global. DOI: 10.1107/S160053681103296X/pv2432sup1.cif

Structure factors: contains datablock(s) I. DOI: 10.1107/S160053681103296X/pv2432Isup2.hkl

Additional supplementary materials:  crystallographic information; 3D view; checkCIF report

## Figures and Tables

**Fig. 1.**
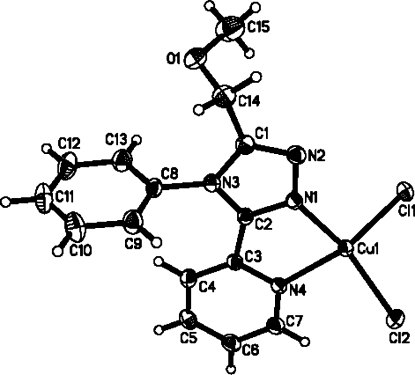
The molecular structure of the title compound with the atomic labels; displacement ellipsoids are shown at 30% probability level.

## supplementary crystallographic information

### Comment

The coordination chemistry of 1,2,4-triazoles as ligands has been widely studied (Klingele & Brooker 2003; Rubio *et al.*, 2011). Some 1,2,4-triazole compounds show biological activities (Isloor *et al.*, 2009). We report here the crystal structure analysis of the title compound.

In the title complex (Fig. 1), copper(II) atom is coordinated by two N atoms of a 3-(methoxymethyl)-4-phenyl-5-(2-pyridyl)-4*H*-1,2,4-triazole and two chloride anion atoms, and exhibits a highly distorted square-planar geometry (Ren *et al.*, 2006) with N1–Cu1–N4 and Cl1–Cu1–Cl2 angles 79.86 (8) and 98.65 (3)°, respectively, while the Cl–Cu–N angles fall in two distinct categories with values 95.26 (6), 98.75 (6), 150.56 (6) and 152.04 (6)°. The pyridyl ring (N4/C3–C7) is twisted by 9.4 (2)° with respect to the triazole ring. The phenyl ring is oriented at approximately right angles (84.66 (8)°) with respect to the triazole ring.

### Experimental

To a warm solution of 3-methoxymethyl-4-phenyl-5-(2-pyridyl)-4*H*-1,2,4-triazole (0.532 g, 2 mmol) in  ethanol (20 ml), CuCl_2_.2H_2_O (0.340 g, 2 mmol) was added. The filtrate was left to stand at room temperature for several days. The title compound crystallized as a green product which was collected and a single crystal suitable for X-ray diffraction was selected.

### Refinement

Positional parameters of all the H atoms were calculated geometrically and were allowed to ride on the parent atoms with C—H = 0.93, 0.96 and 0.97 Å, for aryl, methyl and methylene type H-atoms, respectively, with Uĩso~(H) = 1.2 or 1.5 times U~eq~(C).

### Crystal data

**Table:**

[CuCl_2_(C_15_H_14_N_4_O)]	*F*(000) = 1624
*M**_r_* = 400.74	*D*_x_ = 1.585 Mg m^−^^3^
Orthorhombic, *P**b**c**a*	Mo *K*α radiation, λ = 0.71073 Å
Hall symbol: -P 2ac 2ab	Cell parameters from 9999 reflections
*a* = 16.6512 (11) Å	θ = 2.5–23.3°
*b* = 11.2056 (7) Å	µ = 1.63 mm^−^^1^
*c* = 17.9966 (11) Å	*T* = 296 K
*V* = 3357.9 (4) Å^3^	Plate, green
*Z* = 8	0.15 × 0.13 × 0.12 mm

### Data collection

**Table:**

Bruker APEXII CCD diffractometer	3043 independent reflections
Radiation source: fine-focus sealed tube	2288 reflections with *I* > 2σ(*I*)
graphite	*R*_int_ = 0.052
ω scans	θ_max_ = 25.3°, θ_min_ = 2.3°
Absorption correction: multi-scan (*SADABS*; Sheldrick, 2003)	*h* = −19→19
*T*_min_ = 0.792, *T*_max_ = 0.829	*k* = −13→13
22829 measured reflections	*l* = −21→20

### Refinement

**Table:**

Refinement on *F*^2^	Secondary atom site location: difference Fourier map
Least-squares matrix: full	Hydrogen site location: inferred from neighbouring sites
*R*[*F*^2^ > 2σ(*F*^2^)] = 0.029	H-atom parameters constrained
*wR*(*F*^2^) = 0.068	*w* = 1/[σ^2^(*F*_o_^2^) + (0.0297*P*)^2^ + 1.3987*P*] where *P* = (*F*_o_^2^ + 2*F*_c_^2^)/3
*S* = 1.00	(Δ/σ)_max_ = 0.003
3043 reflections	Δρ_max_ = 0.27 e Å^−^^3^
210 parameters	Δρ_min_ = −0.30 e Å^−^^3^
0 restraints	Extinction correction: *SHELXL97* (Sheldrick, 2008), Fc^*^=kFc[1+0.001xFc^2^λ^3^/sin(2θ)]^-1/4^
Primary atom site location: structure-invariant direct methods	Extinction coefficient: 0.00051 (11)

### Special details

**Table:**

Geometry. All e.s.d.'s (except the e.s.d. in the dihedral angle between two l.s. planes) are estimated using the full covariance matrix. The cell e.s.d.'s are taken into account individually in the estimation of e.s.d.'s in distances, angles and torsion angles; correlations between e.s.d.'s in cell parameters are only used when they are defined by crystal symmetry. An approximate (isotropic) treatment of cell e.s.d.'s is used for estimating e.s.d.'s involving l.s. planes.
Refinement. Refinement of *F*^2^ against ALL reflections. The weighted *R*-factor *wR* and goodness of fit *S* are based on *F*^2^, conventional *R*-factors *R* are based on *F*, with *F* set to zero for negative *F*^2^. The threshold expression of *F*^2^ > σ(*F*^2^) is used only for calculating *R*-factors(gt) *etc*. and is not relevant to the choice of reflections for refinement. *R*-factors based on *F*^2^ are statistically about twice as large as those based on *F*, and *R*- factors based on ALL data will be even larger.

### Fractional atomic coordinates and isotropic or equivalent isotropic displacement parameters (Å^2^)

**Table:**

	*x*	*y*	*z*	*U*_iso_*/*U*_eq_	
Cu1	0.050004 (18)	0.14725 (3)	0.291744 (16)	0.03113 (11)	
Cl1	−0.00195 (5)	0.07124 (6)	0.39349 (4)	0.0465 (2)	
Cl2	0.04038 (5)	0.33855 (6)	0.32076 (4)	0.0497 (2)	
N1	0.11465 (12)	0.00815 (17)	0.25962 (11)	0.0307 (5)	
N2	0.15782 (13)	−0.08048 (19)	0.29457 (12)	0.0363 (5)	
N3	0.17649 (12)	−0.09025 (18)	0.17273 (11)	0.0320 (5)	
N4	0.05062 (13)	0.17404 (17)	0.17923 (12)	0.0315 (5)	
C1	0.19497 (15)	−0.1381 (2)	0.24098 (15)	0.0360 (6)	
O1	0.22057 (13)	−0.34971 (18)	0.22443 (12)	0.0537 (6)	
C2	0.12619 (14)	0.0011 (2)	0.18771 (13)	0.0288 (6)	
C3	0.08684 (14)	0.0871 (2)	0.13891 (14)	0.0298 (6)	
C4	0.08315 (17)	0.0841 (2)	0.06246 (14)	0.0409 (7)	
H4	0.1081	0.0233	0.0359	0.049*	
C5	0.04137 (18)	0.1740 (3)	0.02592 (16)	0.0471 (8)	
H5	0.0372	0.1734	−0.0256	0.057*	
C6	0.00641 (18)	0.2635 (3)	0.06654 (16)	0.0433 (7)	
H6	−0.0209	0.3251	0.0429	0.052*	
C7	0.01245 (16)	0.2607 (2)	0.14301 (15)	0.0390 (7)	
H7	−0.0111	0.3218	0.1703	0.047*	
C8	0.21233 (15)	−0.1229 (2)	0.10221 (14)	0.0336 (6)	
C9	0.27888 (17)	−0.0607 (3)	0.07879 (16)	0.0484 (8)	
H9	0.2997	0.0011	0.1074	0.058*	
C10	0.31432 (19)	−0.0914 (3)	0.01219 (18)	0.0623 (9)	
H10	0.3596	−0.0506	−0.0043	0.075*	
C11	0.2831 (2)	−0.1813 (4)	−0.02921 (19)	0.0651 (10)	
H11	0.3071	−0.2013	−0.0742	0.078*	
C12	0.2167 (2)	−0.2431 (3)	−0.00585 (18)	0.0623 (10)	
H12	0.1961	−0.3045	−0.0350	0.075*	
C13	0.17992 (18)	−0.2144 (3)	0.06103 (16)	0.0473 (7)	
H13	0.1349	−0.2558	0.0775	0.057*	
C14	0.25175 (18)	−0.2404 (3)	0.25097 (17)	0.0472 (7)	
H14A	0.3014	−0.2228	0.2250	0.057*	
H14B	0.2643	−0.2486	0.3034	0.057*	
C15	0.1611 (2)	−0.3976 (3)	0.2710 (2)	0.0763 (11)	
H15A	0.1833	−0.4112	0.3195	0.114*	
H15B	0.1424	−0.4718	0.2507	0.114*	
H15C	0.1171	−0.3426	0.2746	0.114*	

### Atomic displacement parameters (Å^2^)

**Table:**

	*U*^11^	*U*^22^	*U*^33^	*U*^12^	*U*^13^	*U*^23^
Cu1	0.03540 (19)	0.03221 (18)	0.02578 (18)	0.00298 (15)	0.00322 (14)	−0.00052 (13)
Cl1	0.0637 (5)	0.0446 (4)	0.0313 (4)	−0.0037 (4)	0.0134 (3)	0.0013 (3)
Cl2	0.0754 (5)	0.0342 (4)	0.0396 (4)	0.0099 (4)	0.0052 (4)	−0.0038 (3)
N1	0.0329 (12)	0.0315 (12)	0.0276 (12)	0.0032 (10)	0.0021 (9)	0.0020 (9)
N2	0.0372 (12)	0.0378 (13)	0.0340 (12)	0.0046 (10)	−0.0014 (10)	0.0035 (10)
N3	0.0321 (12)	0.0328 (12)	0.0309 (12)	0.0015 (10)	0.0033 (10)	0.0003 (10)
N4	0.0355 (12)	0.0314 (12)	0.0275 (11)	0.0026 (10)	0.0021 (10)	0.0027 (9)
C1	0.0340 (14)	0.0364 (15)	0.0377 (16)	0.0016 (13)	0.0009 (12)	0.0006 (13)
O1	0.0589 (13)	0.0432 (12)	0.0591 (14)	0.0147 (11)	0.0039 (11)	−0.0017 (11)
C2	0.0276 (14)	0.0302 (14)	0.0286 (14)	−0.0020 (11)	0.0017 (11)	−0.0014 (11)
C3	0.0305 (14)	0.0300 (14)	0.0290 (14)	−0.0034 (11)	0.0018 (11)	−0.0002 (11)
C4	0.0520 (17)	0.0395 (16)	0.0310 (16)	0.0062 (14)	0.0013 (13)	−0.0033 (13)
C5	0.063 (2)	0.0525 (19)	0.0256 (15)	0.0009 (16)	−0.0030 (14)	0.0052 (13)
C6	0.0492 (18)	0.0427 (17)	0.0381 (17)	0.0049 (14)	−0.0037 (14)	0.0105 (13)
C7	0.0412 (16)	0.0374 (16)	0.0382 (16)	0.0048 (13)	0.0040 (13)	0.0040 (13)
C8	0.0330 (14)	0.0370 (15)	0.0309 (15)	0.0083 (12)	0.0041 (12)	−0.0035 (12)
C9	0.0444 (17)	0.0537 (19)	0.0469 (18)	−0.0072 (15)	0.0098 (14)	−0.0069 (15)
C10	0.049 (2)	0.089 (3)	0.049 (2)	−0.0019 (19)	0.0160 (16)	−0.0052 (19)
C11	0.061 (2)	0.094 (3)	0.040 (2)	0.017 (2)	0.0122 (17)	−0.0105 (19)
C12	0.076 (3)	0.067 (2)	0.044 (2)	0.005 (2)	−0.0070 (18)	−0.0258 (17)
C13	0.0486 (18)	0.0480 (18)	0.0452 (19)	−0.0026 (15)	0.0009 (14)	−0.0070 (15)
C14	0.0394 (17)	0.0500 (19)	0.0521 (19)	0.0116 (15)	−0.0024 (14)	0.0048 (15)
C15	0.065 (2)	0.062 (2)	0.102 (3)	0.000 (2)	0.017 (2)	0.007 (2)

### Geometric parameters (Å, °)

**Table:**

Cu1—N1	1.981 (2)	C5—H5	0.9300
Cu1—N4	2.047 (2)	C6—C7	1.380 (4)
Cu1—Cl1	2.1969 (7)	C6—H6	0.9300
Cu1—Cl2	2.2121 (7)	C7—H7	0.9300
N1—C2	1.311 (3)	C8—C9	1.375 (4)
N1—N2	1.378 (3)	C8—C13	1.376 (4)
N2—C1	1.315 (3)	C9—C10	1.380 (4)
N3—C2	1.350 (3)	C9—H9	0.9300
N3—C1	1.375 (3)	C10—C11	1.356 (5)
N3—C8	1.449 (3)	C10—H10	0.9300
N4—C7	1.331 (3)	C11—C12	1.371 (5)
N4—C3	1.356 (3)	C11—H11	0.9300
C1—C14	1.496 (4)	C12—C13	1.388 (4)
O1—C15	1.403 (4)	C12—H12	0.9300
O1—C14	1.414 (3)	C13—H13	0.9300
C2—C3	1.459 (3)	C14—H14A	0.9700
C3—C4	1.377 (3)	C14—H14B	0.9700
C4—C5	1.390 (4)	C15—H15A	0.9600
C4—H4	0.9300	C15—H15B	0.9600
C5—C6	1.371 (4)	C15—H15C	0.9600
			
N1—Cu1—N4	79.86 (8)	C7—C6—H6	120.5
N1—Cu1—Cl1	98.75 (6)	N4—C7—C6	122.7 (3)
N4—Cu1—Cl1	152.04 (6)	N4—C7—H7	118.7
N1—Cu1—Cl2	150.56 (6)	C6—C7—H7	118.7
N4—Cu1—Cl2	95.26 (6)	C9—C8—C13	121.9 (3)
Cl1—Cu1—Cl2	98.65 (3)	C9—C8—N3	118.2 (2)
C2—N1—N2	109.3 (2)	C13—C8—N3	119.9 (2)
C2—N1—Cu1	114.52 (16)	C8—C9—C10	119.0 (3)
N2—N1—Cu1	135.85 (16)	C8—C9—H9	120.5
C1—N2—N1	105.3 (2)	C10—C9—H9	120.5
C2—N3—C1	104.8 (2)	C11—C10—C9	119.9 (3)
C2—N3—C8	128.4 (2)	C11—C10—H10	120.0
C1—N3—C8	126.3 (2)	C9—C10—H10	120.0
C7—N4—C3	118.3 (2)	C10—C11—C12	121.1 (3)
C7—N4—Cu1	126.09 (18)	C10—C11—H11	119.5
C3—N4—Cu1	115.22 (16)	C12—C11—H11	119.5
N2—C1—N3	111.0 (2)	C11—C12—C13	120.3 (3)
N2—C1—C14	125.8 (2)	C11—C12—H12	119.9
N3—C1—C14	123.2 (2)	C13—C12—H12	119.9
C15—O1—C14	112.8 (3)	C8—C13—C12	117.8 (3)
N1—C2—N3	109.5 (2)	C8—C13—H13	121.1
N1—C2—C3	119.3 (2)	C12—C13—H13	121.1
N3—C2—C3	131.2 (2)	O1—C14—C1	113.0 (2)
N4—C3—C4	122.1 (2)	O1—C14—H14A	109.0
N4—C3—C2	110.6 (2)	C1—C14—H14A	109.0
C4—C3—C2	127.2 (2)	O1—C14—H14B	109.0
C3—C4—C5	118.5 (3)	C1—C14—H14B	109.0
C3—C4—H4	120.7	H14A—C14—H14B	107.8
C5—C4—H4	120.7	O1—C15—H15A	109.5
C6—C5—C4	119.4 (3)	O1—C15—H15B	109.5
C6—C5—H5	120.3	H15A—C15—H15B	109.5
C4—C5—H5	120.3	O1—C15—H15C	109.5
C5—C6—C7	118.9 (3)	H15A—C15—H15C	109.5
C5—C6—H6	120.5	H15B—C15—H15C	109.5

## References

[bb1] Bruker (2005). *APEX2* and *SAINT* Bruker AXS Inc., Madison, Wisconsin, USA.

[bb2] Isloor, A. M., Kalluraya, B. & Shetty, P. (2009). *Eur. J. Med. Chem.* **44**, 3784–3787. 10.1016/j.ejmech.2009.04.038 19464087

[bb3] Klingele, M. H. & Brooker, S. (2003). *Coord. Chem. Rev.* **241**, 119–132.

[bb4] Ren, X. M., Ni, Z. P., Noro, S., Akutagawa, T., Nishihara, S., Nakamura, T., Sui, Y. X. & Song, Y. (2006). *Cryst. Growth Des.* **6**, 2530–2537.

[bb5] Rubio, M., Hernández, R., Nogales, A., Roig, A. & López, D. (2011). *Eur. Polym. J.* **47**, 52–60.

[bb6] Sheldrick, G. M. (2003). *SADABS* University of Göttingen, Germany.

[bb7] Sheldrick, G. M. (2008). *Acta Cryst.* A**64**, 112–122. 10.1107/S0108767307043930 18156677

